# Patient informed choice in the age of evidence‐based medicine: IVF patients’ approaches to biomedical evidence and fertility treatment add‐ons

**DOI:** 10.1111/1467-9566.13581

**Published:** 2022-11-11

**Authors:** Manuela Perrotta, Josie Hamper

**Affiliations:** ^1^ Department of People and Organisations Queen Mary University of London London UK

**Keywords:** add‐on treatment, evidence, evidence‐based medicine, fertility patients, informed choice, IVF

## Abstract

With the increasing offer of fertility treatment by a largely privatised sector, which has involved the proliferation of treatment add‐ons lacking evidence of effectiveness, In‐Vitro Fertilisation (IVF) patients are expected to make informed choices on what to include in their treatment. Drawing on interviews with 51 individuals undergoing fertility treatment, this article explores patients’ approaches to medical evidence interpretation and its role in their decisions to include add‐ons. While most IVF patients share understandings of what counts as medical evidence, our findings show how their approaches also differ. Our analysis focuses on how patients negotiate the notion of medical evidence and its relation to other forms of experience or knowledge. We present four different approaches to evidence in IVF: (1) delegating evaluations of evidence to experts; (2) critically assessing available evidence; (3) acknowledging the process of making evidence; and (4) contextualising evidence in their lived experience of infertility. We suggest that patients’ choice to include add‐ons is not due to a lack of information on or understanding of evidence, but rather should be interpreted as part of the complexity of patients’ experiences of infertility.

## INTRODUCTION

Medical sociology literature has shown that infertility is a social process that needs to be understood within sociocultural contexts (Leyser‐Whalen et al., 2018). Whereas not all infertile individuals seek treatment (Passet‐Wittig & Greil, 2021), the medicalisation of infertility has introduced the notion that this condition can be treated and accelerated the spread of In‐Vitro Fertilisation (IVF) as the normalised solution to involuntary childlessness (Greil et al., 2010).

Despite the growth of IVF worldwide, its success rates remain relatively low. For instance, in the United Kingdom, the average birth rate per embryo transferred was 23% in 2018 (HFEA, 2020a). In addition, IVF treatment is expensive and public funding is very limited, even in countries with strong public health systems. In England, in 2018, only 35% of IVF treatments were NHS‐funded, while the remaining 65% were self‐funded treatments provided through fully private or NHS clinics. Although medical consumerism is not new to the NHS landscape (Mold, 2010), fertility care is considered a highly commercialised sector due to its high level of privatisation and its ability to attract venture capital and private equity investment (Van de Wiel, 2020).

The experience of infertility, with its emotional and financial burden, is characterised by several challenging choices for patients (Mounce et al., 2022), including: whether to start IVF treatment (Leyser‐Whalen et al., 2018) or consider other less invasive alternatives such as intra‐uterine insemination (Bahadur & Homburg, 2019); which provider to choose (Marcus et al., 2005); and when to stop treatment (Peddie et al., 2005). In recent years, patients have been faced with additional choices related to what their fertility treatment should include (Perrotta & Hamper, 2021). Many additional treatments, known as ‘add‐ons’ in the British public debate, are offered on top of a standard IVF cycle, with the aim of increasing the chances of having a baby. These include a variety of tests, treatments and clinical interventions (see Cochrane, 2021) and their cost can vary from a few to several hundred pounds (Van de Wiel et al., 2020). Patients’ encounters with add‐ons can take various forms as there are significant differences in what clinics offer. Some clinics include add‐ons as part of their standard treatment or offer them as part of a trial, while others charge patients (Perrotta & Hamper, 2021). In addition, add‐ons are sometimes sought by patients through their own research or can be suggested by consultants (Lensen et al., 2021).

Treatment add‐ons have been heavily criticised in the medical literature for the lack of robust evidence of their safety and effectiveness (Cochrane, 2021; Harper et al., 2017) and for the poor quality of information offered to patients on fertility clinic websites (Van de Wiel et al., 2020). Notwithstanding, the number of available add‐ons has proliferated in recent years, and a recent survey showed that over 70% of UK patients have included them in their treatment (HFEA, 2019).

Both in the medical and public debate on add‐ons, concerns have focussed on the offering of unproven treatments to vulnerable patients deemed willing to accept, and who often request, expensive treatments in the hope that they might increase their chances of having a baby. A recent Australian study (Lensen et al., 2021) suggests that patients place high importance on scientific evidence and their decision to include add‐ons in their treatment might be due to poor information about the lack of evidence supporting their use, their risks and benefits.

To ‘ensure that patients can make informed choices about their treatment’ (HFEA, 2020b), in 2017, the HFEA (the UK fertility regulator) introduced a traffic light system (HFEA, 2022) that offers information about the effectiveness and safety of the most widely used add‐ons (currently 12) on the basis of their independent assessment of the available evidence. The traffic light system reflects the dominant understanding of acceptable medical evidence as the outcome of randomised‐controlled trials (RCT) only (Perrotta & Geampana, 2020). According to this system, a green light is assigned to an add‐on when more than one quality RCT proves the effectiveness of that treatment, an amber light indicates conflicting evidence and a red light specifies that there is no evidence of effectiveness. At the time of writing, none of the add‐ons reviewed have a green light, five have an amber light, and seven have a red light.

Despite the heated debate on the lack of evidence supporting add‐ons, little is known about how patients interpret evidence and the role this has in their decisions about fertility treatment. Drawing on interviews with 51 IVF patients, this article bridges this gap. We show that while most patients share and understand articulated notions of what counts as medical evidence, they also negotiate evidence in relation to other forms of experience or knowledge. We identified four approaches to evidence: (1) delegating evaluations of evidence to experts; (2) critically assessing available evidence; (3) acknowledging the process of making evidence; and (4) contextualising evidence in their lived experience of infertility.

### Patient informed choice in the age of evidence‐based medicine

In the last three decades, the concept of patient choice has gained popularity not only among insurance‐based health systems, where choice is traditionally institutionalised, but also among publicly funded national health systems (Costa‐Font & Zigante, 2016). In the UK, the concept of choice in health‐care provision has been introduced with a twofold aim: driving up the quality of care through competition (Fotaki, 2010) and increasing patient involvement, empowerment and autonomy in their care (Fotaki, 2013). These two aims have different implications for patient choice: a market‐level choice between providers of a service and a choice of what specific treatments (medications, therapies, etc.) will be taken up. Although we acknowledge the complex relations between these two forms of choice, the focus of this article is on how patients make decisions about their treatment options and trajectory.

The British case shows how the discourse of choice has been entwined with the introduction of ‘health consumerism’ (Greener, 2009). In the literature (see Gabe et al., 2015), the latter has been associated with two contrasting views of patients/consumers: on one hand, a neoliberal view that deems consumers as active and rational choosers; on the other hand, a view promoting patient rights that sees them as potential victims open to exploitation and manipulation by the health‐care market. If a neoliberal view has uncritically supported choice policies to empower patients (see Fotaki, 2013), many concerns have been raised by the consequent commodification of health that such a view implies. For instance, many studies have shown that, although patients generally appreciate having a choice, they do not always want to take on the responsibility for making decisions about their medical care (Ford et al., 2003; Henwood et al., 2003). Other studies have underlined the problematic aspects of exercising choice when there is an unequal basis in terms of status, knowledge and ability of judgement on the effectiveness of different types of treatment between patients/consumers and professionals/experts (Toerien et al., 2013).

We argue that the concerns around patient choice need to be understood as tensions between two conflicting movements in medical practice and health policy: evidence‐based medicine (EBM), aiming at offering clinicians the best available evidence about the most adequate treatment for patients (Sackett et al., 1996); and patient‐centred care (PCC), concerned with patient participation in clinical decision‐making by taking into account the patients’ perspective, needs and preferences (Stewart et al., 1995). As others have noted, there is surprisingly little overlap between these two approaches (Bensing, 2000). EBM was introduced as the science of integrating the clinician’s judgement with best research evidence and patients’ values and preferences (Sackett et al., 1996). However, the dominant hierarchy of evidence, which accepts RCT‐based evidence only, devalues the individual patient experience and any other form of knowledge (Greenhalgh et al., 2015). In addition, EBM is unable to integrate the informal, tacit and socially shared knowledge being used by people and their networks of carers in managing their conditions. Consequently, the EBM notion of evidence no longer resonates with patient experiences or perceptions of effectiveness (Broom & Tovey, 2007a). PCC has emerged to highlight the need to offer medical care that is actually centred on the patient, rather than on the disease or the doctor. However, focussing on the individuality of the experience of illness makes it impossible to produce reliable evidence of effectiveness (see Bensing, 2000). In addition, while there is general agreement on the principles of PCC in the literature, there is no consensus regarding who should be the final decision‐maker and how much guidance doctors should offer (Ford et al., 2003).

To address the tensions between EBM and PCC, many notions have been introduced to endorse the active participation of patients in their medical care, including decisions on treatment once an ‘evidence base’ has been identified. For instance, to make an *informed choice,* it is expected that the decision‐maker understands the relevant evidence to make a decision that reflects their values (Marteau, 2009). Similarly, the notion of *evidence‐based patient choice* (Hope, 1999) is an attempt to reconfigure the principles of EBM to engage patient perspectives through the provision of unbiased, good‐quality information (i.e., evidence) in a form that is accessible to the patient. Finally, the various available models of *shared decision‐making* between patients and health‐care professionals (see Bomhof‐Roordink et al., 2019) acknowledge that both professionals and patients have relevant expertise to be considered in questions of medical evidence and what matters most to patients. However, often this framework is practised to support patients in considering options and achieve informed preferences (Elwyn et al., 2012), questioning the actual power of patients and their autonomy in the decision‐making process (Joseph‐Williams et al., 2014).

All these notions tend to privilege biomedical over other forms of knowledge and assume that being able to access quality information is key to the patient empowerment process without considering other relevant individual priorities and personal circumstances (Greenhalgh et al., 2015). When conflict emerges between expert and lay perspectives, patients are expected to accept biomedical knowledge for compliance rather than for choice (Dixon‐Woods, 2001). Lay perspectives are often dismissed due to the presumptive attribution of characteristics such as unreliability, vulnerability and emotional instability to patients, generating what has been defined as an ‘epistemic injustice’ (Carel & Kidd, 2014), to underline the numerous and often subtle ways in which patients may be dismissed in their specific capacity as knowers. In addition, all the notions presented above assume that evidence is available and uncontested, while the extended social science literature on medical evidence has shown how notions of ‘good’ or ‘enough’ evidence are shaped by local contexts, medical specialisms and institutional hierarchies (Broom & Adams, 2012; Timmermans & Berg, 2003). Furthermore, many studies have shown how patient advocates and groups entangle medical evidence and patient experience to become involved in and to shape biomedical research (see Epstein, 2008), fostering understandings of lay expertise (Epstein, 1995), experiential knowledge (Caron‐Flinterman et al., 2005) and experience as evidence (Renedo et al., 2018). Research has explored how patient advocates influence biomedical research by engaging in forms of ‘evidence‐based activism’ (Rabeharisoa et al., 2014). Appropriating EBM language, tools and knowledge, patient organisations successfully participate in the definition of what counts as evidence in the medical discourse in order to produce changes useful for patients themselves.

Notwithstanding this rich literature, little is known about how patients interpret medical evidence and how different understandings of evidence and effectiveness inform their treatment decision‐making. The only notable exception is the work of Broom and Tovey (2007a, 2007b, 2008) on cancer patients’ use of complementary and alternative medicine (CAM). Their work shows how patients using CAM do not reject the value of biomedical evidence, but rather challenge the EBM notion of evidence as an abstracted and depersonalised knowledge that does not provide answers on the effectiveness of treatment in their specific cases. In these studies, patients did not challenge the accuracy of statistics in terms of predicting the percentage of people that would positively react to a certain treatment but claimed that the statistics were not helpful to foresee how they and their bodies would react to the treatment. For instance, knowing that 70% of patients treated would survive would not provide them with any concrete knowledge on their own survival—because as an individual you either are in the group that survives or not. Interestingly, these studies show that patients’ tendency to critically assess evidence and their perception of statistics as unable to predict individual outcomes were higher when patients’ prognoses were negative or ambiguous. The authors underline that this was not viewed as an act of desperation but as the need to engage in critical thinking to promote self‐determination and preserve hope in order to cope with the disease and treatment processes.

Broom and Tovey’s studies show how the contrasting ontological and epistemological approaches in terms of what constitutes not only evidence and knowledge in medicine but also health and illness, enacted by biomedical professionals (who provide core cancer care) and CAM therapists (providing additional treatment), are re‐elaborated and rearticulated in patients’ perspectives. In the case of fertility care, similar contrasting ontological and epistemological approaches emerge inside the same professional community (Geampana & Perrotta, 2022): IVF professionals who offer add‐ons are often deemed to have vested commercial interests in their trade (Harper et al., 2017), while those who are against are seen as hampering medical progress and innovation (Macklon et al., 2019).

In this article, we analyse the narratives of individual patients who have to make informed choices in the context of a highly privatised sector, how they approach biomedical evidence and effectiveness and the role of this evidence in their decision‐making on treatment.

## METHODS

The findings presented in this article emerge from a larger research study carried out in England that considered both fertility professionals’ and patients’ perspectives on the introduction of new treatments and technologies in IVF (Geampana & Perrotta, 2022; Perrotta & Geampana, 2021; Perrotta & Hamper, 2021).

This article draws on interviews with 51 IVF patients, which includes 40 women who were going through IVF, 10 male partners and one female partner. At the time of the interview, 26 participants were undergoing or about to start IVF treatment, 15 were pregnant or had pregnant partners, and 10 had one or more children from IVF. Participants were aged between 29 and 47, with a mean age of 36. The recruitment of IVF patients and partners from across England took place through two routes that enabled us to include a range of patients who were undergoing NHS‐funded treatment, privately funded treatment or a combination of both. Firstly, 31 participants were recruited by research nurses at three participating NHS fertility clinics and interviewed on‐site at these clinics. Secondly, 20 participants were recruited via an online survey (*n* = 314) that was distributed on relevant online social media. In this survey, participants were given the option to leave their contact details to be contacted for further research participation. These interviews were conducted either over the phone, at the participant’s home or in a café setting, depending on the participant’s own preference.

While all recruitment methods were aimed at both men and women, recruiting male fertility patients or partners to participate in an interview proved challenging, which reflects a widely documented difficulty in research on reproduction (Culley et al., 2013). In general, participants spoke from socially or economically privileged positions, which would have facilitated their access to fertility treatment. However, our participant group was heterogeneous in terms of their ability to afford private IVF treatment.

The study was approved by Queen Mary University’s research ethics committee (ID 2015.80a/b), the UK Health Research Authority (REC Ref 17/EM/0218) and locally at the participating fertility clinics. All interview participants were given written information and the opportunity to ask questions about the study before providing written consent to participate in the research. Each interview participant was offered a voucher of £20 for their participation. All identifiable information about participants and the participating fertility clinics has been removed from this article to preserve their anonymity.

Interviews lasted between 30 and 80 min, giving a total of around 40 h of interview recordings, which were professionally transcribed verbatim. The transcripts were coded and analysed following an abductive process (Timmermans & Tavory, 2012), which consists of a combination of careful data analysis with a broad theoretical base. This type of analysis follows the traditional grounded theory recommendation to move back and forth between data and theory iteratively and offers further tools to develop novel theoretical insights emerging from surprising empirical findings. In this study, this involved collecting and assigning codes to all references made in interviews to evidence or proof, as well as indirect references to more abstract conceptualisations of evidence that did not necessarily use these terms. For example, this could include references to the legitimacy of medical knowledge or making treatment decisions based on a strong ‘feeling’ or conviction. The semi‐structured interview design allowed for participants to articulate their views and experiences in their own terms (Brinkman & Kvale, 2015). Additionally, participants’ reflections on how they disengaged from questions of evidence were collected as an important counter narrative to those who took a more proactive stance in these debates.

### Findings: Four approaches to evidence interpretation in IVF

Based on this coding process and an in‐depth reading of the interview transcripts, within the participant group we identified four different approaches to evidence interpretation in IVF: (1) delegating evaluations of evidence to experts; (2) critically assessing available evidence; (3) acknowledging the process of making evidence; and (4) contextualising evidence in patients' lived experience of infertility. Considerations about evidence were raised not only in relation to add‐on tests or treatments in IVF but also in other contexts such as the impact of hormones on cancer risk, the side effects of treatment on the health of IVF babies and the effectiveness of nutritional supplements to improve patients’ reproductive health. Although all these articulations of evidence were included in the analysis process, in this article, we focus here on participants’ decision‐making processes in relation to IVF treatment add‐ons.

### Delegating evaluations of evidence to medical professionals

A large group of participants preferred to delegate evaluations of evidence in IVF to medical professionals. This generally meant that they preferred to follow the advice of their clinic or consultant in relation to treatment decision‐making. Their trust in professionals was often closely attached to an understanding and belief that professionals will only ever act in the patient’s best interest and that their clinic would never offer anything that might cause harm. Patients in this category often had reservations about exploring treatment options beyond the perceived basic, standard or routine IVF procedure, unless there was a very strong case (or evidence, broadly defined) for doing something ‘differently’. Sometimes, this could be a consideration about the cost of add‐ons and their affordability or it could be a strategy for limiting the number of options in an attempt to remove some of the burden of making the ‘right’ decisions. Some of the male partners expressed reservations about directing the treatment, given that it primarily takes place on the female body.

The following quote is typical of participants in this group. In this quote, the participant emphasised that she would not research optional treatments and their evidence‐base in favour of following the clinic’s treatment recommendations. In addition to this, she also noted how following the guidance of her clinic involved a kind of emotional calibration. The ‘people’ that this participant refers to in this quote are the fertility professionals at her clinic:

I’d rather just not know, I’d rather just trust kind of what people are telling me. And if they were worried then I should be worried kind of thing. So I didn’t really, no, I didn’t really look things up. But then I think if, because everything went so smoothly and so quickly that I didn’t really feel like I needed to. Maybe if it hadn’t then I would have been googling.

(Woman, aged 30, pregnant from first embryo transfer)

Notably, this participant was pregnant from her first embryo transfer at the time of the interview and had not been a fertility patient for as long as many of the other participants. Her reflection on whether she would do more independent research into treatment options (‘googling’) if she had been in treatment for longer reflects a tendency within the participant group for patients to become increasingly involved in directing their treatment if they had experienced several failed attempts.

The following participant also articulated her approach to decision‐making as grounded in a relationship of trust with the consultant and the clinic. She explained how she felt most comfortable in following the guidance of the clinical team and specifically made a point of not researching treatment options but accepting the course of treatment that was recommended for her:

I went in trying not to get too personally, it sounds silly, trying not to get personally involved, I tried to keep it quite clinical almost as well, just go in and go right, please tell me, you’re the expert, I’ll trust you, so. I felt like I did kind of trust them and just kind of hoped that it worked. And certainly, when the other consultant spoke to us and she offered us those three extra things [an anticoagulant drug, a culture medium called EmbryoGlue, and time‐lapse embryo imaging], again for me they sounded logical and again I was sort of just kind of hopeful and I hadn’t done much research into what they were. I just kind of trusted them I think. She was very friendly. Very nice.

(Woman, aged 40, pregnant from IVF and had a baby from IVF previously)

Disengaging from the responsibility of making treatment decisions can offer a strategy for maintaining distance from the emotional strain of decision‐making. The previous participant described this as an attempt to keep the treatment process ‘quite clinical’ and thus not become ‘personally involved’. She explained how starting treatment felt like a relief from the strain of trying to conceive previously: ‘Once I was in the IVF process there was a sense of I can relax a little bit, I am with experts and with the top people […] I could put my faith and my trust in them. And I think I did almost completely.’ She noted that she was aware of the limited evidence‐base to support these ‘three extra things’, which she paid for as part of a self‐funded cycle of IVF, but she reiterated throughout the interview that she was willing to follow the advice of the clinical team.

### Critical evaluations of evidence

Another group of participants engaged in what we have termed critical evaluations of evidence. These participants saw themselves as playing a key and active role in assessing the available evidence for treatments that were offered to them, which would often involve doing extensive research drawing on a wide range of (mainly) online resources. The focus and outcome of critical evaluations of evidence varied between participants. Some would reject any additional treatment that was not supported by RCT. Others would emphasise a small but good‐quality evidence‐base or state that if a treatment is based on ‘sound science’ but has simply not been through enough testing, then this can be sufficient to justify its use. This group of participants would often adopt medical concepts and terminology to evaluate and reflect on evidence in IVF, and potentially use this to challenge suggestions or decisions made by fertility consultants.

Participants who approached evidence critically would often draw on their professional knowledge or skills in assessing the evidence for a particular procedure, whether this was being able to navigate the scientific literature or through their familiarity with certain treatments from working in a medical or health‐care profession. One participant noted how their work shaped their ability to critically examine research studies: ‘I guess I’m a bit sceptical about, you know, working a bit with data in my job, you know, I know that it’s possible to make things swing one way or the other and it depends on, you know, the number you have. It depends on the studies.’ (Woman, aged 36, pregnant from first embryo transfer). In this case, critically evaluating evidence is extended to include evaluating the reliability of the studies and study methodologies that produce evidence.

Others were able to access peer‐reviewed articles through their professional work or network, such as an affiliation with a university. Access, in this context, refers both to the ability to physically access journals and articles that might otherwise only be available behind paywalls, but it also captures the ability to interpret and evaluate scientific writing, methods and research. The following participant and her partner, both of whom worked at a university, had been through multiple rounds of failed transfers by the time of the interview, which meant that there had been time for them to become increasingly invested and detailed in their learning:

I think working at university has got benefits and that would be able to access peer reviews, we both work at universities so we’re used to doing that kind of research. So we’re quite well skilled to look. So we’d been doing a lot of research and looking up information from the start.

(Woman, aged 37, preparing to start fourth cycle of IVF, one miscarriage)

She went on to describe how their consultant had recommended endometrial scratch, a procedure carried out before an IVF cycle where the lining of the womb (the endometrium) is ‘scratched’ using a small sterile plastic tube to make it more receptive to an embryo implanting. This add‐on has an amber light on the HFEA traffic system (HFEA, 2022) as there is currently conflicting evidence from RCTs to show that it is effective at improving the chances of having a baby for most fertility patients:

The consultant has suggested that we look at an endometrial scratch prior to the cycle starting. And when we first had a conversation with him there was some evidence to say that it might help, it might not, but there was no randomised trial. Since then, about a week later, the new study came out from New Zealand and that was that.

(Same as above)

This participant saw the report of this study (Lensen et al., 2019) while following ‘the conference in Spain’ in 2018 (i.e., the annual meeting of the European Society of Human Reproduction and Embryology that was held in Barcelona) and then looked up the study, an RCT that shows no significant improvement in the rates of ongoing pregnancy or miscarriage for patients undergoing this treatment.

Whereas some participants perceived RCTs as the definitive source of evidence to support a treatment, a critical approach did not always mean that participants would disregard any treatment that was not supported by similar forms of high‐quality evidence. This approach could also involve risk calculations or evaluating whether there is ‘enough’ evidence to support a particular test or treatment. It could also involve questioning the feasibility or definition of evidence in IVF. The following participant had read a book that offered advice for how to improve egg quality through lifestyle changes, such as nutrition and reducing exposure to environmental toxins, and she reflected on the evidence base for these recommendations:

I followed near enough everything in that book. Because although I know that there’s not *enough* clinical evidence to support some of them there’s *a lot* of clinical evidence and for me I suppose if you look at the things like folic acid, there wasn’t enough clinical evidence for that for many, many years. I just thought I’ll give everything a go. So I’ve done lots of things to change, to try and improve and my second round was much, much better. So we got thirteen eggs.

(Woman, aged 39, preparing for first embryo transfer)

By drawing on the widely familiar and accepted recommendation in the UK for women to take folic acid during early pregnancy and the pre‐conception stage (NHS, 2020), the previous participant rationalises her choice to follow recommendations in IVF that are based on ‘a lot’ but potentially not ‘enough’ clinical evidence. Notably, her references to wanting to ‘give everything a go’ related specifically to lifestyle changes, whereas she was reticent about trying more medicalised forms of treatment.

### The making of evidence

Some participants were concerned with the *definition* of evidence in IVF. These participants had a strong awareness of the many aspects of reproductive biology and IVF that are still unknown within the medical sciences, and they accepted therefore that there may be some experimental aspects to IVF as a whole as well as their individual treatment. For example, one participant situated her willingness to try unproven treatments against a view that ‘the whole thing [IVF] is a bit of a scientific experiment’. Closely connected to this view was an emphasis on innovation, exploration and the development of knowledge in reproductive medicine, which we refer to as the ‘making’ evidence in IVF. This could sometimes involve reflections on the significant barriers to creating high‐quality evidence in IVF, such as the difficulties in coordinating multiple clinics or recruiting a sufficient number of study participants (Perrotta & Geampana, 2021).

Sometimes, participants’ reflections on the making of evidence in IVF were grounded in their own experiences of participating in a research trial as part of their fertility treatment. These participants often perceived themselves as having a personal role in advancing the field of reproductive medicine. For example, one stated that: ‘medicine doesn’t advance unless you try things.’ Participants in this group often reflected on the unknown qualities of IVF and infertility more generally, and how medical research is needed to address significant gaps in knowledge and understanding. This view was often underpinned by experiences of receiving an ‘unexplained infertility’ diagnosis, as demonstrated by the following participant:

I suppose it just feels significant and important to be part of any trialling or testing that is, in one way or the other, whether it’s negative information or positive information, going to contribute to getting more information. I feel like fertility is something we really, really know nothing about. To have had years and years of tests and be a perfectly healthy woman and not have any rhyme or reason or any clue of why I can’t get pregnant. And you know, if the IVF doesn’t work I feel like I’ll still have absolutely no idea why that was.

(Woman, aged 38, preparing for first embryo transfer)

Participants in this group generally placed high value on the potential for them to contribute to knowledge creation and recognised that any outcome of the trial, which the previous participant refers to as ‘negative information or positive information’, is still a valuable contribution.

Some participants had considered including treatment add‐ons that, at the time of the research, were in the initial stages of testing, such as the effect of natural killer cells on embryo implantation. Participants often situated the use of immunological tests and treatments—which are rated red in the HFEA traffic light system (2022)—as controversial based on the reading they had done as well as potential discussions with their own consultants.

The following participant, who was preparing for further treatment after four unsuccessful embryo transfers, reflected on the theory of natural killer cells testing:

[The] theory is that natural killer cells, which are in your blood, all over your body and work to attack viruses and infections, actually if you’ve got too many of them they can attack the embryo because half of it is a foreign body because half belongs to the male and I think that’s the issue. So if the killer cells are too high they, well, they believe that in women where the killer cells are too high, too many of them, that you’re attacking the healthy embryo […] I think it’s quite controversial, I think some people are very opposed about it, [they] think it’s not accurate, so it’s a theory.

(Woman, aged 33, preparing for sixth embryo transfer)

This participant’s emphasis on the ‘theory’ of natural killer cells and the ‘belief’ that is driving investigations in this area constitutes this field of knowledge as uncertain. In the absence of any other explanation for why the embryos transferred had not implanted beyond unexplained infertility, the theory of natural killer cells holds an explanatory potential that is appealing.

### Contextualising evidence in the lived experience of infertility

Some participants situated their understanding of evidence in the wider context of their lived experience of infertility. Although few detailed this contextualisation explicitly, many interviewees inferred the need to consider their interpretation of evidence and effectiveness in relation to their own circumstances and histories. In this way, certain treatments ‘made sense’ to participants for a variety of reasons that are not readily captured within a medical evidence‐base framework.

When patients are called to make choices about treatment in conditions of high uncertainty, as often there are no known causes of infertility and there is a generalised lack of evidence to support treatment (Cochrane, 2021), they have to rely on other forms of knowledge and their previous experience. The following patient reflected on her choice to take aspirin, which is thought to possibly improve implantation rates by increasing blood flow to the uterus but is not currently a general recommendation for IVF patients due to a lack of evidence that it increases pregnancy rates (Gelbaya et al., 2007). Her decision to proceed with this was shaped by her knowledge of other patients’ experiences combined with an evaluation of aspirin as constituting a low risk of harm:

I’m sure it was through one of those sorts of [online] forums that somebody had said about, like their clinic had recommended taking this low grade aspirin, and I just thought well, actually it kind of makes sense, you know, it thins the blood. It was like 75 mg or, it was a tiny, tiny little aspirin. And it really was something where I thought, you know what, if I’m not allergic to it and it’s not really going to cause me any harm, it’s like taking it for a headache, so yeah, that I chose to do.

(Woman, aged 34, had baby from IVF and became an egg donor)

This participant negotiated the lack of evidence to support the use of aspirin in IVF by trivialising this medication in terms of quantity (‘a tiny, tiny little aspirin’) and normalising its use for common ailments (‘it’s like taking it for a headache’). She also noted how the potential benefit of aspirin ‘makes sense’ based on her existing knowledge and understanding of how this medication works (‘it thins the blood’) and thus improves blood circulation.

Patient decision‐making about treatment was often influenced by their experience of infertility, and patients re‐negotiated notions of medical evidence, uncertainty and risk of potential harm on the basis of their personal circumstances. The following patient reflected on how the length of involuntary childlessness contributes to the re‐alignment of possible benefit versus risk:

I’ll give my example because me and my missus were married for eight years before we had a child. Now let’s say we were married for twenty years and we didn’t have a child and then you came up to us and said listen, there’s this new revolutionary medication which will help you but there are these side effects. Now depending what those side effects are and you said listen we haven’t tested it properly on everyone like it’s been 80% tested, it’s safe, there’s a 20% chance it might not be safe and you might have these side effects. The fact that we’ve been married for twenty years and we hadn’t had a child and this could be our last chance, maybe we would be willing to take that risk.

(Man, aged 34, had one baby from IVF and starting further treatment for a second child)

Comparing and reflecting on different treatments, risk categories and types of evidence in light of patients’ lived experiences of infertility was common among patients. The following participant described her evaluation of risk when deciding to pursue, and pay for, the controversial immunological treatment discussed above in the form of steroids:

It’s difficult because like the steroids, obviously they could do harm, but I felt like the dose that I was on it was, you know, there was a risk but I felt like it was a risk I was willing to take […] I knew some of the side effects of the steroids, you know, I knew the risks but I took a cal‐, you know, that was my risk, I took it.

(Woman, aged 35, had a baby from fifth embryo transfer, two miscarriages)

As demonstrated in the previous quote, contextualising evidence in the lived experience of infertility could sometimes involve taking known risks in treatment. This participant had been through several failed cycles of IVF, including miscarriages, which likely shaped her willingness to pursue a riskier treatment option. She recognised that steroids are not supported by rigorous evidence that they improve chances of pregnancy, and that they may even be harmful, but she claimed this risk as one she was willing to take.

## DISCUSSION

As discussed above, several notions have been introduced to conceptualise patient informed choice in the age of evidence‐based medicine. However, all these notions tend to privilege biomedical over other forms of knowledge (Dixon‐Woods, 2001; Greenhalgh et al., 2015) and to reproduce ‘epistemic injustice’ (Carel & Kidd, 2014) by dismissing lay perspectives. Despite this growing literature, with the notable exception of the work of Broom and Tovey (2007a, 2007b, 2008), little is known about patients’ understandings and interpretations of evidence and its role in their treatment choice. In this article, to close this gap, we focus on IVF patients’ approaches to biomedical evidence interpretation and fertility treatment add‐ons. The case of fertility add‐ons is of particular interest as there is disagreement on the use of these treatments within the professional and scientific community due to the lack of evidence of their effectiveness (Harper et al., 2017; Macklon et al., 2019).

By exploring the varied articulations of evidence offered by our interviewees, our analysis offers a nuanced understanding of how patients interpret biomedical evidence and how its role in their treatment decisions is re‐negotiated on the basis of various factors, including their lived experience of infertility. As the extended social science literature on medical evidence has shown, what constitutes ‘good’ or ‘enough’ evidence is shaped by local circumstances (Broom & Adams, 2012; Timmermans & Berg, 2003). Our findings show that patients’ interpretations are similarly shaped by their individual and social circumstances.

Notably, our participant group was heterogeneous in terms of their ability to both afford private IVF treatment and assess evidence. Echoing previous studies (Ford et al., 2003; Henwood et al., 2003), a large group of participants did not want to take on the responsibility for deciding about their medical care and preferred to delegate evaluations of evidence to medical professionals. Although the reasons for choosing to delegate differed considerably, delegation here is a form of active choice similar to what Charis Thompson (2005) defines as ‘agency through objectification’, to underline how patients submit to technical procedures in order to retain some sense of agency and selfhood. Yet it is important to note that there are structural inequalities and barriers to ‘free’ choice, as some patients felt they did not have the agency or the financial resources to direct their treatment decisions. Our study confirms the literature showing how problematic it can be to exercise choice considering the unequal basis in terms of status, knowledge and ability of judgement between patients/customers and professionals/experts (Toerien et al., 2013), but also shows high variations among patients in terms of their ability to negotiate interpretations of evidence and exercise choice.

Although many patients chose to delegate assessment of evidence to medical professionals, most of our participants were included in one of the three additional approaches to evidence interpretations we identified. These approaches show how patients actively and critically engage with the dominant notion of biomedical evidence, offering articulated understandings but negotiating its role in relation to other forms of knowledge and experience. Some patients, often drawing on their professional backgrounds or skills, claimed an active role in treatment choice by doing extensive research to personally assess the available evidence for treatments that were offered to them. Although these participants widely perceived RCTs as the definitive source of evidence to support a treatment, they sometimes questioned what counts as ‘enough’ evidence to support a particular test or treatment considering the limited evidence available now. Similarly, another group of participants challenged the definition of evidence in IVF by acknowledging the high level of uncertainty that characterises reproductive medicine and the barriers to creating high‐quality evidence in IVF (Perrotta & Geampana, 2021). Like the previous group, these interviewees were highly aware of what counts as evidence and of the lack of evidence for specific treatments, but underlined the appealing explanatory potential of the theory behind some treatments when infertility is unexplained. Finally, a third approach was for participants to contextualise their understanding of evidence in their lived experience of infertility, their own circumstances and histories. Acknowledging the high uncertainty that characterises infertility and the generalised lack of evidence to support treatment add‐ons (Cochrane, 2021), these patients claimed the need to rely on other forms of knowledge and their previous experience to make choices. Also, in this case, the potential choice of unproven or risky treatment was not due to poor information or a lack of understanding of what counts as evidence, but was explained by the need to take incremental risk when previous treatment did not work.

As in the case of cancer patients using CAM (Broom & Tovey, 2007a, 2007b, 2008), the last three approaches to evidence interpretation show that most IVF patients do not devalue biomedical evidence in principle, but rather feel they have to put their treatment choice into perspective by critically assessing available evidence (in a context where robust evidence is largely lacking), situating knowledge production in the uncertain field of reproductive medicine, and contextualising their understanding of evidence (and attitude to risk) in their lived experience of infertility. In addition, echoing Broom and Tovey’s work that shows how cancer patients were less orientated towards biomedical evidence when their prognoses were negative or ambiguous, IVF patients showed higher flexibility in their interpretations of evidence when there was no explanation for their infertility or they had been through many unsuccessful cycles.

## CONCLUSION

The findings presented in this article have profound implications for medical sociology, fertility care practice and policy and the wider fields of medical practice and health policy.

Firstly, our analysis of patient informed choice in the age of evidence‐based medicine underscores the gap between EBM and PCC, and offers insights to bridge this gap. We defy reproducing ‘epistemic injustice’ (Carel & Kidd, 2014) by investigating how patients negotiate the notion of medical evidence and its role in their treatment choices. Our analysis shows that patients are neither active and rational choosers nor victims open to exploitation and manipulation by the health‐care market. Rather, they are called to make complex treatment choices in highly uncertain conditions where biomedical knowledge is unable to provide explanations and there is a lack of robust evidence. We challenge the view of patients as vulnerable and willing to accept and even request unproven, expensive treatment as a desperate act to increase their chances of having a baby. Our findings show that some patients did not want to take on the responsibility for deciding about their treatment and intentionally chose to delegate evaluations of evidence to medical professionals, while others actively engaged with situated interpretations of the available evidence in consideration of their personal circumstances and history of infertility. We argue that further social science research addressing the tensions between EBM and PCC in other fields of medicine is crucial to develop an integrated approach.

Secondly, we challenge the assumption that patients’ decisions to include add‐ons in their treatment are due to a lack of understanding of what counts as evidence or because they are not well informed (Lensen et al., 2021). Our findings highlight patients as a heterogeneous group with different needs and priorities, and so have crucial implications for fertility care practitioners and policy‐makers. Although the provision of unbiased, good‐quality information (i.e., evidence) in a form that is accessible to patients is necessary, it is not enough to support patient choice. As fertility is a social experience, other relevant individual priorities and personal circumstances should be acknowledged and considered by integrating patients’ perspectives, needs and priorities in the debate and in the production of evidence itself (Perrotta & Geampana, 2021). In addition, alternative strategies should be explored to protect those patients who claim the right to delegate the responsibility for their health care to professionals.

Thirdly, in an increasingly privatised contemporary health‐care landscape, we argue that the dynamics presented in this article are not specific to the fertility sector only, and our analysis may be helpful to address the complexity of exercising informed choice in a variety of medical fields. Understanding how patients approach medical evidence interpretation in general is key to revealing potential effects of the privatisation of health care and should be at the forefront of future research to support patients in making informed choices in the resulting market.

## AUTHOR CONTRIBUTIONS

**Manuela Perrotta**: Conceptualization (lead); Formal analysis (supporting); Funding acquisition (lead); Methodology (lead); Project administration (lead); Supervision (lead); Writing – original draft (equal); Writing – review & editing (equal). **Josie Hamper**: Conceptualization (supporting); Data curation (lead); Formal analysis (lead); Investigation (lead); Writing – original draft (equal); Writing – review & editing (equal).

## Data Availability

The data that support the findings of this study are available on request from the corresponding author. The data are not publicly available due to privacy or ethical restrictions.
